# Amplification of the Gene Ontology annotation of Affymetrix probe sets

**DOI:** 10.1186/1471-2105-7-159

**Published:** 2006-03-20

**Authors:** Enrique M Muro, Carolina Perez-Iratxeta, Miguel A Andrade-Navarro

**Affiliations:** 1 Ontario Genomics Innovation Centre, Ottawa Health Research Institute, 501 Smyth Rd, Ottawa ON, K1H 8L6, Canada

## Abstract

**Background:**

The annotations of Affymetrix DNA microarray probe sets with Gene Ontology terms are carefully selected for correctness. This results in very accurate but incomplete annotations which is not always desirable for microarray experiment evaluation.

**Results:**

Here we present a protocol to amplify the set of Gene Ontology annotations associated to Affymetrix DNA microarray probe sets using information from related databases.

**Conclusion:**

Predicted novel annotations and the evidence producing them can be accessed at Probe2GO: . Scripts are available on demand.

## Background

The biological interpretation of DNA microarray experiments to measure gene expression requires contrasting the detected patterns of gene expression (obtained, for example, using a clustering algorithm) with the functions of the genes that correspond to those patterns. Because of the high number of genes interrogated by this technique, human interpretation is difficult and a number of computational methods have been developed. Since there are a number of resources that contain annotations of genes with terms of the Gene Ontology (GO) describing the related pathways, processes, or cellular locations of the gene products [1], many of these computational methods use GO annotations attached to genes as a means to describe microarray experiments (for example, [2-4]).

In the case of the Affymetrix DNA microarrays, probe set annotations with GO terms are provided and updated on a regular basis (NetAffx; [5]). However, although the number of probe sets covered with gene information and GO annotations tends to increase from release to release, we noticed upon analyses of particular experiments that these annotations could be improved. On the one hand, we observed that some probe sets were linked to a database entry annotated with GO terms that could be directly transferred. On the other hand, we found helpful to have a supply of predicted GO terms obtained by inference from the content of different databases, increasing the level of gene description, and, in some cases, providing functional information to genes not yet annotated.

With this idea in mind, we have used two strategies to amplify the GO annotations associated to Affymetrix probe sets: (1) direct transfer of GO annotations from related databases; and (2) inferred transfer for the predictions that requires examining associations between GO terms and annotations from one or more linked databases.

## Results and discussion

**Figure 1:**
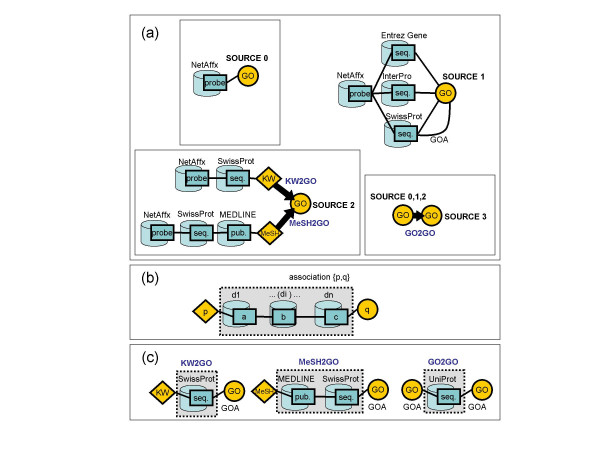
**Scheme of the process followed to retrieve GO terms associated to Affymetrix probe sets**. Cylinders are databases, boxes are entries, diamonds and circles are attached properties. Plain lines are direct links, whereas arrows indicate a fuzzy relation of inclusion in the direction of the arrow. See text for details of those databases. **(a) Sources of GO terms**. Source 0: NetAffx. Source 1: links from NetAffx to other databases annotated with GO terms. Sources 2 and 3 use inference from properties associated to linked entries. Source 2 consists of GO terms derived from SwissProt keywords (KW2GO mapping) and MeSH terms (categories A, C, D, and G) from MEDLINE (MeSH2GO mapping). Source 3 consists of GO terms derived from sources 0, 1, and 2, by a mapping between GO terms (GO2GO mapping). **(b) General schema for the definition of a mapping**. A fuzzy mapping is computed by analysis of co-occurrences of the values of a property "p" attached to a database *d*_1 _entry (left) and another property "q" attached to database *d*_*n *_entry (right) via any number of intermediate databases. **(c) Mappings used in this work**: KW2GO, MeSH2GO, and GO2GO. See Methods for details.

We obtain GO annotations from four types of sources (see Figure 1 and Methods). Source 0 consists of the GO terms originally associated by NetAffx to a probe set. Source 1 consists of GO terms found in database entries linked to the probe set by NetAffx. Source 2 consists of predicted GO terms obtained from inferred relations between GO terms and terms from other controlled vocabularies present in database entries linked to the probe set. Source 3 consists of predicted GO terms inferred from GO terms already derived from sources 0, 1, or 2, for a probe set.

We have evaluated our method for the probe sets of both the MOE430 and HG-U133 Affymetrix DNA microarrays for the mouse and the human genomes, respectively, using the NetAffx release from December 2004. In the case of HG-U133, the set of annotations produced (union of sources 1, 2 and 3) consists of 154,425 GO term annotations that cover 26,124 probe sets out of a total of 44,760 in the microarray, including annotations for 2,987 probe sets without any GO term (see Table 1). These 154,425 GO term annotations have a recall of the annotations given by NetAffx (source 0) of 98.1% (see Table 2). The figure of precision respect source 0 (76.9%) does not necessarily indicate that the system performs badly as the main use of this system is to produce new annotations. Some of those are already well known but not found in NetAffx and some are predicted. An example of the latter case is probe 219280_at from chip HG-U133 representing the uncharacterized protein WD repeat domain 9 that was annotated with the GO term "nucleus GO:0005634" by our system (source 2). This protein corresponds to SwissProt entry Q9NSI6, which is annotated with two keywords describing its domain content: "Bromodomain" and "WD repeats". According to the inferred relations described above, the SwissProt keyword "Bromodomain" is included in the GO term "nucleus". The association accounts for the fact that bromodomains are found in many chromatin associated proteins. This type of associations between SwissProt keywords and protein cellular locations have been used before for prediction [6]. Here we introduce a system that expands the concept by including in general all Gene Ontology terms and all databases possibly linked (directly or indirectly) to a probe set.

In total, the method produced 35,608 new annotations for the probe sets of the HG-U133 Affymetrix DNA microarray (Table 1). For this calculation we have also considered the structure of the ontology (directed acyclic graph): a term derived from source n was not considered new if it was an ancestor of any other term found in sources m ≤ n. In absolute numbers, source 1 was clearly the one producing the most of the new annotations, with sources 2 and 3 producing one order of magnitude less of annotations. We note that probe sets lacking any GO annotation in the NetAffx table did very rarely receive new annotations from source 2, and did not receive any new annotation from source 3 as they derive from other GO annotations. Source 2 and source 3 annotations applied, therefore, mostly to probe sets with already some degree of previous annotation.

For reference, the NetAffx release used for this analysis (December 2004) added 27,228 new annotations respect to the previous one of June 2004. The application of our protocol to that previous release produced a total of 58,493 new annotations of which 16,166 were included in the NetAffx December 2004 release. We note that those 16,166 were mostly from source 1 (only one from source 2 and five from source 3), which agrees to the fact that NetAffx annotations are derived by direct transfer from other databases [5].

To test the validity of the predicted new GO terms, we manually evaluated random selections from the 35,608 new annotations for the probe sets of the HG-U133 Affymetrix DNA microarray mentioned above for each type of source. As it could be expected, we found that the new terms derived from source 1 (direct transfer from other database) were by far the most reliable (29 valid terms out of 29 tested; 100%). The terms from source 2 were less reliable (15 valid terms out of 27 tested; 56%) with source 3 (inferred from another GO term) producing the least reliable set of new annotations (14 valid terms out of 30 tested; 47%). GO annotations were taken as valid if they were supported by experimental evidence or by reasonable sequence features. We illustrate this with two examples.

A positive example is the association to probe set 201034_at of the source 3 GO term GO:0015629 ("actin cytoskeleton", that includes actin cytoskeleton-associated complexes) by inference from the GO-term "structural constituent of cytoskeleton", which was associated to this probe by NetAffx. Probe set 201034_at, according to NetAffx, refers to the *ADD3 *gene, that encodes the adducin 3 (gamma) protein. Adducins are cytoskeletal actin-binding proteins [7] and then this prediction counted as valid.

A negative example is the association to probe set 36829_at of the source 2 GO term GO:0003677 ("DNA binding") by inference from the keyword "Transcription regulation", which was found in the SwissProt entry O15534 (for the human Period circadian protein 1) linked to the probe set by NetAffx. However, although "Transcription regulation" often implies an interaction with DNA, this particular protein has not been proven to directly interact with DNA, and it does not contain domains indicating such function. It regulates transcription by forming complexes with several DNA-binding proteins such as the cryptochrome 1 protein (CRY1; [8]). Therefore this prediction counted as invalid.

We have established a public web server (Probe2GO; [9]) coded in Perl with a back-end based in a local MySQL database server. Users can retrieve amplified GO annotations for a given probe set (or a list of them). The server allows tracing the path of evidence followed to derive these annotations, which should allow users to select annotations to their desired level of reliability.

In order to support computational methods that rely on the complete set of GO annotations for the probe sets in a chip, those can be obtained following the links indicated in the Probe2GO entry page. These files will be updated with every NetAffx release.

Following the recommendation of the GO consortium to indicate an evidence code for all GO annotations, we note that since the association between a probe set and a gene is subject to the interpretation of the related genomic information and this can change over time (as described in [10]), all our GO annotations should receive the IEA (inferred from electronic annotation) evidence code according to [11]: "Annotations transferred from database records, if not reviewed by curators".

## Conclusion

We have presented a resource to expand the GO annotations of Affymetrix probe sets that we have developed motivated by our needs to expand probe set annotations as much as possible.

Our future goal is to add to our protocol other databases and links that could serve as sources of additional evidence to improve the specificity of the predictions (for example by analysis of the literature deposited in MEDLINE), as well as to apply the protocol to an expanding number of microarrays.

## Methods

We used four sources of GO terms (Figure 1a). **Source 0 **consists of the GO terms associated to the probe set as provided by NetAffx. **Source 1 **consists of the GO terms obtained from database entries linked to the probe set by NetAffx. We use Entrez Gene [12], InterPro [13], SwissProt [14], and the GO terms associated by the Gene Ontology Annotation (GOA [15]) to the UniProt entries linked to SwissProt. **Source 2 **consists of predicted GO terms obtained from inferred relations between GO terms and other controlled vocabularies using a mapping from SwissProt keywords to GO terms (KW2GO) and a mapping from MeSH terms in MEDLINE entries [16] to GO terms (MeSH2GO). **Source 3 **consists of predicted GO terms inferred from GO terms already derived from sources 0, 1, or 2, using a mapping between GO terms (GO2GO).

The three mappings (KW2GO, MeSH2GO, GO2GO) were obtained following a procedure previously described [17,18]. The association between a property, p, attached to a database entry *d*_1 _(Figure 1b left) and another property, q, attached to a database entry *d*_*n *_(Figure 1b right) can be obtained by examining the instances where entries with those properties in *d*_1 _and *d*_*n *_can be connected via any number of intermediate databases. The two mapped properties can be, for example, keywords in SwissProt entries and GO terms associated to SwissProt entries.

First, we count the number of pairs of values for p and q that are in connected entries. Then, given two particular values for those properties, p_1 _and q_1_, we define an association score of inclusion of p_1 _in q_1 _as the number of {p_1_, q_1_} pairs divided by the total of pairs containing p_1_: a value close to 1 means that p_1 _implies q_1_. The support of this relation of inclusion is defined by the number of {p_1_, q_1_} pairs. The larger the support, the more reliable is the relation.

For example, we examined the SwissProt keywords and the GO terms associated to SwissProt entries. We found 43 SwissProt entries that had both the keyword "Bromodomain" and the GO term "nucleus". The support for the relation between these two terms is therefore 43. We also counted 49 entries containing the keyword "Bromodomain" (irrespective of whether they were associated to the GO term "nucleus" or not). The value of inclusion of the "Bromodomain" SwissProt keyword in the GO term "nucleus" is then 43/49 = 0.877, according to this mapping; this supports the notion that the SwissProt keyword suggests the GO term. In this work, we consider only relations both with scores of inclusion ≥ 0.8 and support ≥ 5 (following [18]).

The three mappings used, KW2GO, MeSH2GO, and GO2GO (Figure 1c), were obtained in the following way: **KW2GO **was obtained using all pairs {Keyword, GO term} related via entries in SwissProt. The assignments of GO terms to SwissProt entries were obtained from the GOA database [15]. **MeSH2GO **was obtained using all pairs {MeSH term, GO term} related via entries in SwissProt. We considered only MeSH categories A, C, D, and G, as the ones that contain information that relates to GO. MeSH terms were assigned to SwissProt entries using the linked MEDLINE entries, which are annotated with MeSH terms. To ensure that we were using MeSH terms reliably related to the corresponding gene, only MeSH terms present in at least three MEDLINE entries pointed by a SwissProt entry were used. This prevented many wrong associations originating from MEDLINE entries that refer to multiple genes and proteins (data not shown). **GO2GO **was obtained using all pairs {GO term, GO term} related via entries in UniProt.

Additionally, we eliminated all direct transfers and relations involving certain GO and MeSH terms lacking content (for example, the GO term "biological process unknown") or incorrect in the context of the analysis (for example, the GO term "chloroplast" while analyzing mammals). The updated list of terms is accessible from the web site.

## Authors' contributions

All authors participated in the conception of Probe2GO. MA coordinated the project. EM implemented Probe2GO. CP computed inference associations using data mining methods. EM and CP evaluated the method. All authors participated in the drafting of the manuscript, and read and approved the final manuscript. MA was previously known as Miguel A. Andrade.

## Figures and Tables

**Table 1:** Coverage of Affymetrix probe sets with GO terms

	**MOE430**				**HG-U133**			
	**Probe sets**		**GO terms**		**Probe sets**		**GO terms**	

**Source**	**Total**	**New**	**Total**	**New**	**Total**	**New**	**Total**	**New**

0	22051	-	117761	-	23206	-	121031	-
1	24735	2756	144952	29095	26115	2978	147036	28298
2	9468	3	32332	1105	13917	9	41949	3705
3	12395	0	21792	3621	12657	0	20191	3605
1,2,3	24738	2759	149749	33821	26124	2987	154425	35608

The total of probe sets in MOE430 and HG-U133 chips are 45,101 and 44,760, respectively. New: number of probe sets or annotations not covered by any source with a lower number. Source 0 are the annotations given by NetAffx; 1 are annotations taken from linked databases; 2 are annotations implied by association to terms in linked databases; 3 are annotations implied by GO terms in 0, 1, or 2. 1,2,3 are the union of the annotations of sources 1, 2, and 3.

**Table 2:** Recall and precision of amplified GO annotations respect to NetAffx given GO annotations.

	**MOE430**		**HG-U133**	
**Source**	**Recall**	**Precision**	**Recall**	**Precision**

1	98.3%	79.9%	98.1%	80.7%
2	25.4%	92.7%	30.0%	86.6%
3	13.3%	72.0%	12.2%	73.4%
1,2,3	98.4%	77.4%	98.1%	76.9%

**Recall **is defined as the percentage of NetAffx annotations (source 0) that are found in the Probe2GO annotations. **Precision **is the percentage of Probe2GO annotations that are provided by NetAffx (source 0). 1,2,3: union of the annotations of sources 1, 2, and 3.
